# Adaptation and validation of the revised two-factor study process questionnaire (R-SPQ-2F) for tertiary physical education contexts in China

**DOI:** 10.3389/fspor.2025.1682949

**Published:** 2025-11-07

**Authors:** Shengying Tang, Peizi Han, Dandan Chen, Rui Zhang

**Affiliations:** 1 School of Education (Normal School), Dongguan University of Technology, Dongguan, China; 2 School of Management, Beijing Sport University, Beijing, China

**Keywords:** learning approaches, reliability, validity, confirmatory factor analysis, factor structure, higher education

## Abstract

**Introduction:**

Improving the quality of physical education (PE) is vital for countering global physical inactivity and establishing lifelong active lifestyles. Research indicates that students' approaches to learning (SAL) are a key determinant of educational quality. However, a validated, psychometrically sound instrument to measure these approaches specifically within the unique context of non-PE tertiary students is currently lacking.

**Purpose:**

This study aimed to validate the Revised Two-Factor Study Process Questionnaire (R-SPQ-2F), an instrument developed by Biggs and widely utilized across various academic disciplines to measure tertiary students' learning approaches, within the specific context of physical education (PE).

**Methods:**

Data were collected from 492 Chinese undergraduates spanning multiple academic fields. Confirmatory factor analysis was performed on six competing models using two estimators, WLSMV and MLMV.

**Results and discussion:**

Results indicated that a first-order two-factor model comprising ten items measuring the deep approach (DA) subscale and eight items measuring the surface approach (SA) subscale exhibited good fit to the data. Reliability analyses further confirmed strong internal consistency, with coefficients of 0.947 for the overall structural model, 0.928 for the DA subscale, and 0.855 for the SA subscale. These findings provided evidence that the adapted 18-item R-SPQ-2F was a valid and reliable tool for measuring Chinese undergraduates' learning approaches in PE contexts. By establishing a valid measure of PE learning approaches, this study provided a foundation for designing targeted interventions that bridge the improvement of PE learning approaches with active lifestyle promotion. Future validation in diverse PE settings and student populations is recommended to strengthen its utility for such health-promotion initiatives.

## Introduction

In modern society, physical inactivity has emerged as one of the major threats to human health (1, 2). Despite the well-established evidence that regular physical activity serves as a protective factor in preventing and managing non-communicable diseases while enhancing mental health, altering people's sedentary behaviour remains a global challenge (3, 4). Consequently, physical education (PE) has been endowed with an important mission: teaching people how to maintain active lifestyles across the lifespan and in diverse settings, a goal that has gained worldwide consensus.

In China, PE as a compulsory course of general studies has been extended to tertiary institutions, with requirements stipulating no fewer than 144 h of PE courses for university students in their first two years and at least 108 h for junior college students. This policy aims to establish a solid foundation for lifelong active living during the final phase of formal education. However, many studies have found that problems remain in the learning quality of physical education experienced by Chinese undergraduates, such as inadequate teacher-student interaction (5), low levels of student engagement (6), and insufficient physical exercise (China Youth Network, 2023). Against the backdrop of declining physical fitness among Chinese higher education students (7, 8), improving student learning quality is of paramount importance for advancing China's PE reform and achieving the objectives of the “Healthy China” initiative.

Research on higher education over recent decades has indicated that students' approaches to learning (SAL), which connotes the tendencies that individuals adopt in the face of learning materials and strategies for dealing with learning contents, is an important factor affecting the learning quality (9–12). Based on the interplay of motives (the reasons or goals behind why students learn) and strategies (particular activities, methods, or processes that students engage in during learning), students' learning approaches can be categorized into two distinct orientations: the deep approach (DA) and the surface approach (SA) (11, 13, 14). A surface approach to learning is primarily motivated by extrinsic factors or fear of failure, with learners adopting strategies that involve minimal time and effort to merely meet assessment requirements. As noted by Biggs (15), this approach often “refers to activities of an inappropriately low cognitive level, which yields fragmented outcomes that do not convey the meaning of the encounter” (p.60). In contrast, the deep approach to learning is driven by an intrinsic interest in the subject matter of the task, with learners prioritizing strategies to maximize understanding. Biggs (16) described this orientation as focusing “on underlying meaning rather than on the literal aspects of the task” (pp.6–7), with the intention to “extract maximum meaning from it” (p.7). Previous studies have demonstrated positive associations between the deep approach to learning and learning outcomes (17), including higher academic achievement (18), enhanced cognitive and personality development (19), and sustained engagement in learning (20, 21). Therefore, fostering deep learning among students has emerged as a central focus in contemporary educational research.

In the field of physical education, recent years have witnessed a growing emphasis on deepening the understanding of the nature of movement and physical education (22–26). However, deep learning in physical education is discussed predominantly from dimensions such as meaning-making (27, 28), intrinsic motivation (29, 30), or self-determination (31), with relatively less attention paid to students' learning approaches. In fact, learning approaches denote the overall patterns of behaviours and attitudes through which students seek meaning, stimulate motivation, and acquire learning strategies within a given learning setting (16). Measuring these approaches can provide critical insights for the overall evaluation of learning outcomes in physical education. Moreover, research has pointed out that students do not always adopt the learning approach that is best suitable to bring about the desired learning outcomes without guidance and instructions (32). This highlights the importance of diagnosing students' problems in learning approaches as a novel and valuable perspective for improving the quality of PE learning and fostering lifelong physical education habits. By addressing these approach-related issues, educators can better support students in constructing deep, meaningful learning experiences that align with the long-term goals of physical education.

Among the various self-report questionnaires designed to measure students' learning approaches, including the Revised Approaches to Studying Inventory (RASI), the Approaches and Study Skills Inventory for Students (ASSIT), and the Revised Two-Factor Study Process Questionnaire (R-SPQ-2F), the R-SPQ-2F developed by Biggs et al. (33) is common and simple to employ. It has been widely adapted for studies across diverse higher education subjects, including business (34), osteopathy (35), anatomy & physiology (36), and engineering (37).

While developing a completely new instrument could offer maximum content specificity for PE context, adapting a validated and widely utilized instrument like the R-SPQ-2F holds distinct strengths due to its superior analytical utility and psychometric foundation. Utilizing an adapted instrument ensures that findings maintain crucial theoretical continuity with the extensive body of existing research on learning approaches—an advantage that is particularly valuable for PE research, which currently lacks a unified framework for studying SAL. This continuity not only enables cross-disciplinary comparisons but also enhances the generalizability and interpretability of results, helping to situate PE learning patterns within the broader SAL theory and address the gap in PE-specific SAL research identified earlier. Nevertheless, to the best of our knowledge, the application of the R-SPQ-2F in the field of PE and sports remains highly limited, with only the study by Tannoubi et al. (38) exploring its relevance. In their research, Tannoubi et al. developed the Physical Learning Process Questionnaire (PE-SPQ), a new instrument that has a significant correlation with the factors of the R-SPQ-2F but is specifically used to measure the learning approaches of students majoring in PE and sports. Tannoubi et al. argued that PE and sports students are required not only to master theoretical knowledge but also to carry out practical training. This unique learning process, which integrates theoretical concerns with physical performance, suggests that the learning approaches of PE students differ from those in other disciplines. As a result, Tannoubi et al. concluded that the development of a specified instrument was necessary for PE and sports majors.

Notably, students who are not majoring in PE and sports have limited exposure to formal sports theory courses, and their PE learning is primarily centred on basic physical movement skills. This physical practice-oriented learning process not only differs from that of PE majors but also presents distinct characteristics compared to the learning approaches in other academic disciplines. Given these distinctions, a critical question arises: Can the original R-SPQ-2F or its adapted version be used to measure the PE learning approaches of the non-PE and sports population?

With this in mind, this study examined the applicability of the R-SPQ-2F in PE learning scenarios for non-PE and sports undergraduates in China. Similar to studies in other disciplines, the present study mainly focuses on examining the psychometric properties of the R-SPQ-2F, regarding its factor structure, validity, and reliability. By systematically evaluating the instrument's performance in this unique educational context, the study aims to establish a scientific foundation for the potential adaptation and application of the R-SPQ-2F in PE teaching for non-professional populations.

## Literature review

The R-SPQ-2F developed by Biggs et al. (33) is a self-report questionnaire with a five-point Likert scale. It contains 20 items divided into two main scales, DA and SA. Each of the scales consisted of two 5-item subscales: Deep Motivation (DM) and Deep Strategy (DS) for DA and Surface Motivation (SM) and Surface Strategy (SS) for SA. Biggs et al. (33) tested the psychometric properties of the R-SPQ-2F with two hypothesized models. The first was a first-order four-factor model with four subscales formulated as latent constructs to test the instrument's structure from the items level. The test was based on a sample of 495 undergraduate students from various disciplines across each year of study from one university in Hong Kong. The CFA results supported the unidimensionality of the four subscales, with SRMR = 0.058, CFI = 0.904, and Cronbach alpha values for each subscale in the instrument being 0.62 for DM, 0.63 for DS, 0.72 for SM, and 0.57 for SS. The second was a first-order two-factor model, with four subscales as indicators of two higher-order latent factors, DA and SA. This model tested the dimensionality of the instrument from the scale level. The results also indicated a good fit with SRMR = 0.015, CFI = 0.992, and a negative correlation (-0.23) between DA and SA. The reliability check was reported as 0.73 and 0.64 for DA and SA, respectively. Biggs et al. concluded that the R-SPQ-2F can be used in a two-factor second-order form (33).

Since its development, the R-SPQ-2F questionnaire has been translated into various languages and used in different countries worldwide. Researchers tested not only the validity of the instrument in different linguistic and cultural contexts (12, 32, 39–41) but also its applicability in different disciplines.

Fryer et al. (34) conducted a factor analytic study to test the construct validity of the R-SPQ-2F based on a sample of 269 mixed major (Management and Commerce) students in a Japanese four-year university. The CFA model of the R-SPQ-2F was initially poorly fitting (CFI = 0.78, TLI = 0.73, RMSEA = 0.063), but after items 3, 8, 12, 19 were removed from the questionnaire, the 16-item instrument achieved an improved fit (CFI = 0.88, TLI = 0.85, RMSEA = 0.051). At the dimension level, the four-factor structure achieved a perfect fit (CFI = 1, TLI = 1, RMSEA = 0), but the reliability of the four sub-scales was unsatisfactory. Especially, the correlation between DA and SA is positive (*r* = 0.30), rather than the negative correlation obtained within the Hong Kong and Australian contexts (33, 42). The findings suggested that the R-SPQ-2F performed differently in the Japanese four-year higher education setting than it did in other cultural contexts. In particular, the factor structure and reliability of surface learning methods are low, which may be related to the characteristics of the Japanese educational environment. Fryer et al. called for more student learning theory (SLT) research, longitudinal studies in particular, in the Japanese context to better understand the dynamic relationship between Japanese students' learning approaches and their perception of the learning environment, arguing that this would help develop instruments that are more appropriate to the Japanese cultural context.

Vaughan (35) explored and tested the validity of the R-SPQ-2F in the Australian osteopathy student population (*n* = 197). Satisfactory fit supported a first-order two-factor model (DA and SA) by removing item 3 “My aim is to pass the course while doing as little work as possible”, CFI = 0.995, TLI = 0.995, SRMR = 0.071, RMSEA = 0.013, *χ*^2^(df = 151) = 156.09. According to Vaughan, Modification Indices (MI) had conflicting suggestions on which factor (Deep or Surface) item 3 should be loaded to. Vaughan provided three possible explanations for this situation: First, this item may not accurately indicate a deep or surface learner in an osteopathy student population. Second, this item contained two ideas within one item. Third, its expression can lead to different understandings. Vaughan believed the results provided evidence for the validity of the scores derived from the R-SPQ-2F and this 19-item version could be used to evaluate the learning process of osteopathy students in Australia.

Johnson et al. (36) triangulated qualitative and quantitative results to test the validity of the R-SPQ-2F when the instrument is administered to undergraduate Anatomy & Physiology (A&P) students. The central research question was whether R-SPQ-2F could effectively distinguish between deep and surface learning approaches for A&P students at a research university in the southeastern United States. The study found that the R-SPQ-2F was not able to group students by deep and surface approaches to learning in the context of an A&P course. Six interviews demonstrated that many students' learning approaches fall somewhere between depth and surface, particularly the 'surface leading to deep' approach, which is inconsistent with the R-SPQ-2F dichotomy. The CFA result showed that although the internal consistency (McDonald's omega scores of 0.798 for DA and 0.788 for SA) of the instrument is higher than the Cronbach's alpha value (0.73 for DA and 0.64 for SA) reported by Biggs et al. (33), the fit index values (CFI = 0.801, TLI = 0.777, RMSEA = 0.069, SRMR = 0.072) were only “acceptable”, which indicated that the model fit of the R-SPQ-2F in this sample is not ideal. Johnson et al. pointed out that one possible explanation for the issues observed with the R-SPQ-2F in their study was the lack of recognition of the “achieving” approach to learning (that is, students believe that memorization is necessary while at the same time wanting to understand the material). Another possible reason was that some items in the questionnaire have word interpretation issues, misalignment with the curriculum background, and compound items, which affect their applicability in specific disciplinary contexts. Like other studies, this study recommended that the validity of the R-SPQ-2F in the target population should be tested before use. For populations for which the overall model fit of the instrument to the data is poor, the instrument should be revised or redeveloped to more accurately measure students' learning approaches in specific disciplinary contexts.

From the above studies, it becomes evident that in addition to the recognized cultural sensitivity, the R-SPQ-2F is also discipline-sensitive. This proves, to a certain extent, the necessity of this study. Meanwhile, the existing research also provides a reference for this study.

## Materials and methods

This study was conducted at Dongguan University of Technology (DGUT), China, and received ethical approval from the Academic Ethics Committee of the institution where the first author is affiliated.

### Participants

Participants of this study were year-one and year-two non-PE and sports undergraduates from DGUT. Like other Chinese universities, students at DGUT are required to choose a physical education course each semester in the first two years. However, students have been informed that participating in this research was not part of the teaching plan and would not affect the evaluation of their PE courses. Students gave their consent to participate when they voluntarily submitted the questionnaire. This study used an electronic questionnaire. Participants scanned the QR code provided by their PE teachers in class and answered anonymously.

The survey was distributed in 18 PE classes of swimming, yoga, Taekwondo, football, basketball, and aerobics, with 20–40 students in each class. A total of 492 students responded to the questionnaire, with an average completion time of 276.83 s. Of these participants, 216 were male and 276 were female; 84 were liberal arts majors, 92 were science majors, 316 were engineering majors; 260 were in year 1, and 232 were in year 2.

### Instrumentation

Since the R-SPQ-2F was developed in English, the questionnaire was first translated independently by two associate professors who are native Chinese speakers. One is from the English department, majoring in Chinese-English translation; the other is from the PE department and has experience of studying in English-speaking countries. Then, two translated versions were compared and discussed. During this process, two key issues were fully considered. The first one is semantic equivalence (43), that is, the meaning of items is the same in both English and Chinese versions. The second one is curriculum context/alignment (33, 36), that is, items should be tailored as closely as possible to the PE context. Of these two, it is more difficult and time-consuming to combine items with the characteristics of the PE curriculum to enable students, as Biggs et al. (33) noted, “to reply in connection with the particular course, module, or program in question, rather than to studying generally” (p.145).

A typical example was the translation and wording modification of the concept “rote learning”, an important indicator of SA in the questionnaire. Although the term rarely appeared in the study of physical education, it is undeniable that in PE learning, there are indeed some situations of “exercising/practicing without understanding” (which are typical characteristics of rote learning) (20, 44). For example, in learning fundamental movement skills, students may learn through mechanical practice rather than understanding movement principles. This way of learning has caused a prominent problem in Chinese physical education: students are still not competent and confident enough to take part in a range of physical activities or to move properly and effectively within a wide variety of environments after 12 years of physical education (45). For another example, many studies advocating meaningful PE have pointed out that many forms of school-based physical education failed to inspire students to seek and reflect on “personal significance” (46) in physical activities, thus failing to promote children's continued participation in physical activity through the integration of physical activity into life (28, 62). Based on these, this study held that the concept of “learning things by rote rather than understanding” is also applicable to describe the approach of PE learning, but considering the uniqueness of PE with more emphasis on “physical practice”, expressions focusing on “memorization” were replaced with “practice”. For example, item 11 was adapted as *I find I can get by in most sports assessments by practicing key sections rather than trying to understand them*, and item 20 as *I find the best way to pass examinations is to practice by rote over and over again*.

After initial agreement on the wording and expression, the questionnaire was translated back into English by a foreign teacher from the English department. This foreign teacher has lived in China for nearly 10 years, married a Chinese colleague, and has a profound understanding of Chinese and Western cultures and languages. Finally, according to the experts' opinions on cultural differences, some minor changes were made to the questionnaire, and the final draft was confirmed (see Table 1).

**Table 1:** The back-translated version of the R-SPQ-2F questionnaire adapted in this study.

Item No.	Statement on approaches to learning PE
1	I find that at times studying PE gives me a feeling of deep personal satisfaction.
2	I find that I have to do enough work on a PE or sports topic so that I can form my own conclusions before I am satisfied.
3	My aim is to pass the PE course while doing as little work as possible.
4	I only study seriously what's given out in PE class or in the PE course outlines.
5	I fell that virtually any PE and sports topic can be highly interesting once I get into it.
6	I find most new PE and sports topics interesting and often spend extra time trying to obtain more information about them.
7	I do not find my course very interesting so I keep my work to the minimum.
8	I learn some contents of PE and sports by rote, going over and over them until I know them by heart even if I do not understand them.
9	I find that studying PE and sports topics can at times be as exciting as a good novel or movie.
10	I test myself on important topics until I understand them completely.
11	I find I can get by in most sports assessments by practicing key sections rather than trying to understand them.
12	I generally restrict my study on PE and sports to what is specifically set as I think it is unnecessary to do anything extra.
13	I work hard at my studies because I find the learning material interesting.
14	I spend a lot of my free time finding out more about interesting topics which have been discussed in my PE class.
15	I find it is not helpful to study PE or sports topics in depth. It confuses and wastes time, when all you need is a passing acquaintance with topics learned.
16	I believe that lecturers shouldn't expect students to spend significant amounts of time studying material everyone knows won't be examined.
17	I come to PE classes with questions in mind that I want answering.
18	I make a point of looking at most of the suggested materials that go with the lectures.
19	I see no point in learning material which is not likely to be in the PE or sports examination.
20	I find the best way to pass examinations is to practice by rote over and over again.

In this study, as in Biggs' original version, responses to each item were also rated on a five-point Likert scale: 1 = This item is *never or only rarely* true of me; 2 = This item is *sometimes* true of me; 3 = This item is true of me about *half the time*; 4 = This item is *frequently* true of me; 5 = This item is *always or almost always* true of me).

### Data analysis

A confirmatory factor analysis (CFA) was conducted using Mplus version 8.3 to verify whether the two-factor solution obtained by Biggs et al. (33) adequately fitted the data for this study. To check the fit of the confirmatory factor structure, a variety of indices commonly employed in related studies were used, including Chi-Square, Tucker–Lewis index (TLI), Comparative Fit Index (CFI), Root Mean Square Error of Approximation (RMSEA), and Standardized Root Mean Square Residual (SRMR).

Given the need for robust parameter estimation and model fit for ordinal categorical data (Likert-type scale), two complementary robust estimation methods were adopted for the CFA: Maximum Likelihood Mean-Variance Adjusted (MLMV) and Weighted Least Square Mean-Variance Adjusted (WLSMV). These methods, both enhancements over traditional Maximum Likelihood (ML) and Weighted Least Squares (WLS), are superior for small-to-moderate sample sizes and relax the problematic assumption of multivariate normality (47). Critically, they offer distinct and complementary modelling strengths. MLMV treats the ordinal scores as arising from a latent continuous variable and provides robust (Satorra-Bentler) corrections for nonnormality (48). Conversely, WLSMV is based on the robust modelling of discrete (ordinal) using a polychoric correlation matrix, which is considered more accurate when dealing with fewer response categories (49). While both estimators have theoretical limitations—MLMV relies on the assumption of underlying continuous variables, and WLSMV's standard error estimation can be unstable with very small sample sizes (50)—this combined strategy mitigates these risks; specifically, given the moderate-to-large sample size of this study (*N* = 492), the practical impact of WLSMV's small-sample limitation is minimized. More importantly, by utilizing both methods, a crucial cross-validation strategy across the assumptions of latent continuity (MLMV) and inherent discreteness (WLSMV) was implemented, thereby ensuring maximal robustness of the model fit and parameter estimates.

Building on this methodological foundation, existing research, this study employed MLMV and WLSMV estimation methods to conduct CFA on a total of six models, including four hypothetical models (M1-M4) and two modified models (M5-M6). The specifics were as follows:

1. Model 1 was derived from the classical model hypothesized by Biggs et al. (33), comprising four latent variables (DM, DS, SM, SS), each composed of five observed indicators, with a correlation specified between DM and SM.
2. Model 2 was a full first-order model proposed by Socha et al. (32), in which the four factors were pairwise correlated. Socha et al. argued that this model facilitates the detection of potential misfits that may occur in second-order models.
3. Model 3 was the item-level second-order model assumed by Biggs et al. (33), consisting of two first-order factors (DA and SA) and four second-order factors (DM, DS, SM, and SS).
4. Model 4 was the first-order two-factor model proposed by Biggs et al. (33), with DA and SA as latent variables, each containing 10 observed indicators.
5. Model 5 was a modification of Model 4, with one observed indicator removed from SA.
6. Model 6 was a first-order two-factor model developed by further modifying Model 5, comprising 18 observed indicators (8 assigned to SA).

## Results

Models 1–3 were four-factor models. CFA results from the two robust estimation methods (MLMV and WLSMV) showed that all models generated error messages due to “non-positive definite latent variable covariance matrices”. Specifically, WLSMV parameter estimation revealed standardized inter-latent correlations exceeding 1.0 for certain variables (detailed in Table 2).

**Table 2:** Correlation coefficient matrices of latent variables in models 1–3.

Latent variables	Model 1				Model 2			
	DS	DM	SM	SS	DS	DM	SM	SS
DM	1.000				1.000			
DS	**1** **.** **031**	1.000			**1** **.** **053**	1.000		
SM	−0.596	0.000	1.000		−0.568	−0.482	1.000	
SS	0.000	0.000	**1** **.** **138**	1.000	−0.529	−0.437	**1** **.** **054**	1.000
	Model 3							
	DM	DS	SM	SS	DA	SA		
DM	1.000							
DS	1.053	1.000						
SM	−0.517	−0.482	1.000					
SS	−0.509	−0.437	**1** **.** **074**	1.000				
DA	**1** **.** **023**	**1** **.** **030**	−0.506	−0.498	1.000			
SA	−0.495	−0.499	**1** **.** **044**	**1** **.** **029**	−0.484	1.000		

Bold values indicate standardized inter-latent correlations greater than 1.000.

As noted by Hooper et al. (51), a perfect correlation (*r* = 1.0) indicates complete overlap in the measured content of two constructs, while a correlation exceeding 1.0 suggests a lack of discriminant validity between constructs. When the MLMV estimation method was used, a singular sample covariance matrix of the dependent variables was observed, indicating the presence of linear dependencies in the model (47). Since a positive definite variance/covariance matrix is a prerequisite for obtaining valid CFA solutions (47), fit indices and factor loading data for Models 1–3 were not reported in the main text. Relevant materials are available upon request to the first author.

This study attempted to modify the four-factor models by removing variables that overlapped with others, based on system prompt information and variable correlation coefficients. However, the modification results were unsatisfactory, with some models exhibiting abnormal negative factor loadings. Take Model 1 as an illustration: when item 11 was removed according to system prompts and WLSMV estimation was applied, the factor loadings of items 13 and 17 to DM were −0.441 and −0.375, respectively, while those of items 15 and 19 to SM were −0.660 and −0.552, respectively. These results indicated that the four-factor models suffered from over-factorization of data in this study, accompanied by non-positive definiteness of the covariance matrix. Therefore, such model frameworks should be rejected.

Model 4, a two-factor model with 20 items, was found to yield a positive definite covariance matrix when WLSMV parameter estimation was applied. However, the system prompted that the correlation between item 11 (*I find I can get by in most sports assessments by practicing key sections rather than trying to understand them*) and item 20 (*I find the best way to pass examinations is to practice by rote over and over again*) was 1. In terms of goodness-of-fit indices, although certain indicators of Model 4 (CFI = 0.916, TLI = 0.905, SRMR = 0.65) reached the generally accepted “good fit” threshold (52, 53), the RMSEA value of 0.093 only met the “mediocre fit” criterion (between 0.08 to 0.10) proposed by MacCallum et al. (54) and the chi-square statistic was significantly high (*χ*^2^ = 881.061, *df* = 161). This indicated that the model required further modification. When MLMV estimation was employed, the system flagged redundant variables in Model 4, leading to non-convergence of iterations. Based on these findings, a modified model, Model 5, was constructed and subjected to CFA analysis.

Model 5 was constructed using an item reduction strategy. As items 11 and 20 in the SA exhibited perfect collinearity (Pearson *r* = 1.0), one item was removed, yielding a 19-item two-factor model. Given the mathematical equivalence of those two items, eliminating either resulted in statistically identical estimates for structural and measurement parameters. Thus, this study did not differentiate between the two item deletion schemes. CFA results showed Model 5 converged to an admissible solution, with multiple goodness-of-fit indices indicating favourable model-data fit (see Table 3 for details). The inter-factor correlations between DA and SA were estimated as −0.493 (WLSMV) and −0.453 (MLMV).

**Table 3:** Goodness of fit indices for model 5.

Fix index	Without item 11/20	
	WLSMV	MLMV
Chi-Square	694.077	367.331
Degrees of Freedom	151	151
Number of Free Parameters	96	58
*P*-Value	0.0000	0.0000
CFI	0.935	0.913
TLI	0.926	0.901
RMSEA	0.085	0.053
SRMR	0.056	0.057

Given that the RMSEA value (0.085) obtained through WLSMV estimation was still slightly above the conventional cutoff value of 0.08, a systematic test was conducted on the estimation parameters of Model 5. Item 8 (*I learn some contents of PE and sports by rote, going over and over them until I know them by heart even if I do not understand them*) was identified as potentially problematic. The standardized factor loading for item 8 (0.277 for WLSMV, 0.328 for MLMV) was significantly lower than other items (see Table 4). R-squared values were 0.077 (WLSMV) and 0.107 (MLMV), respectively, indicating insufficient construct explanatory power for this item. In such a situation, item deletion was recommended (47, 55). Accordingly, item 8 was excluded from the SA in this study, thereby forming Model 6 for subsequent analysis. This iterative optimization process aimed to ensure optimal model fit and construct reliability.

**Table 4:** Standardized CFA results of model 5.

Item No.	R-Square		Residual Variance		Factor Loading			
					DA	SA	DA	SA
	WLSMV	MLMV	WLSMV	MLMV	WLSMV		MLMV	
SPQ1	0.595	0.539	0.405	0.461	0.771	-	0.734	-
SPQ2	0.442	0.417	0.558	0.583	0.665	-	0.645	-
SPQ3	0.314	0.252	0.686	0.748	-	0.560	-	0.502
SPQ4	0.476	0.391	0.524	0.609	-	0.690	-	0.626
SPQ5	0.517	0.455	0.483	0.545	0.719	-	0.674	-
SPQ6	0.668	0.603	0.332	0.397	0.817	-	0.777	-
SPQ7	0.641	0.434	0.359	0.566	-	0.800	-	0.659
SPQ8	0.077	0.107	0.923	0.893	-	0.277	-	0.328
SPQ9	0.524	0.466	0.476	0.534	0.724	-	0.682	-
SPQ10	0.542	0.509	0.458	0.491	0.736	-	0.714	-
SPQ12	0.521	0.462	0.479	0.538	-	0.722	-	0.679
SPQ13	0.732	0.664	0.268	0.336	0.856	-	0.815	-
SPQ14	0.666	0.619	0.334	0.381	0.816	-	0.786	-
SPQ15	0.339	0.322	0.661	0.678	-	0.582	-	0.567
SPQ16	0.427	0.357	0.573	0.643	-	0.653	-	0.598
SPQ17	0.483	0.379	0.517	0.621	0.695	-	0.616	-
SPQ18	0.490	0.409	0.510	0.591	0.700	-	0.639	-
SPQ19	0.392	0.342	0.608	0.658	-	0.626	-	0.585
SPQ20	0.375	0.303	0.625	0.697	-	0.613	-	0.550

**Figure 1:**
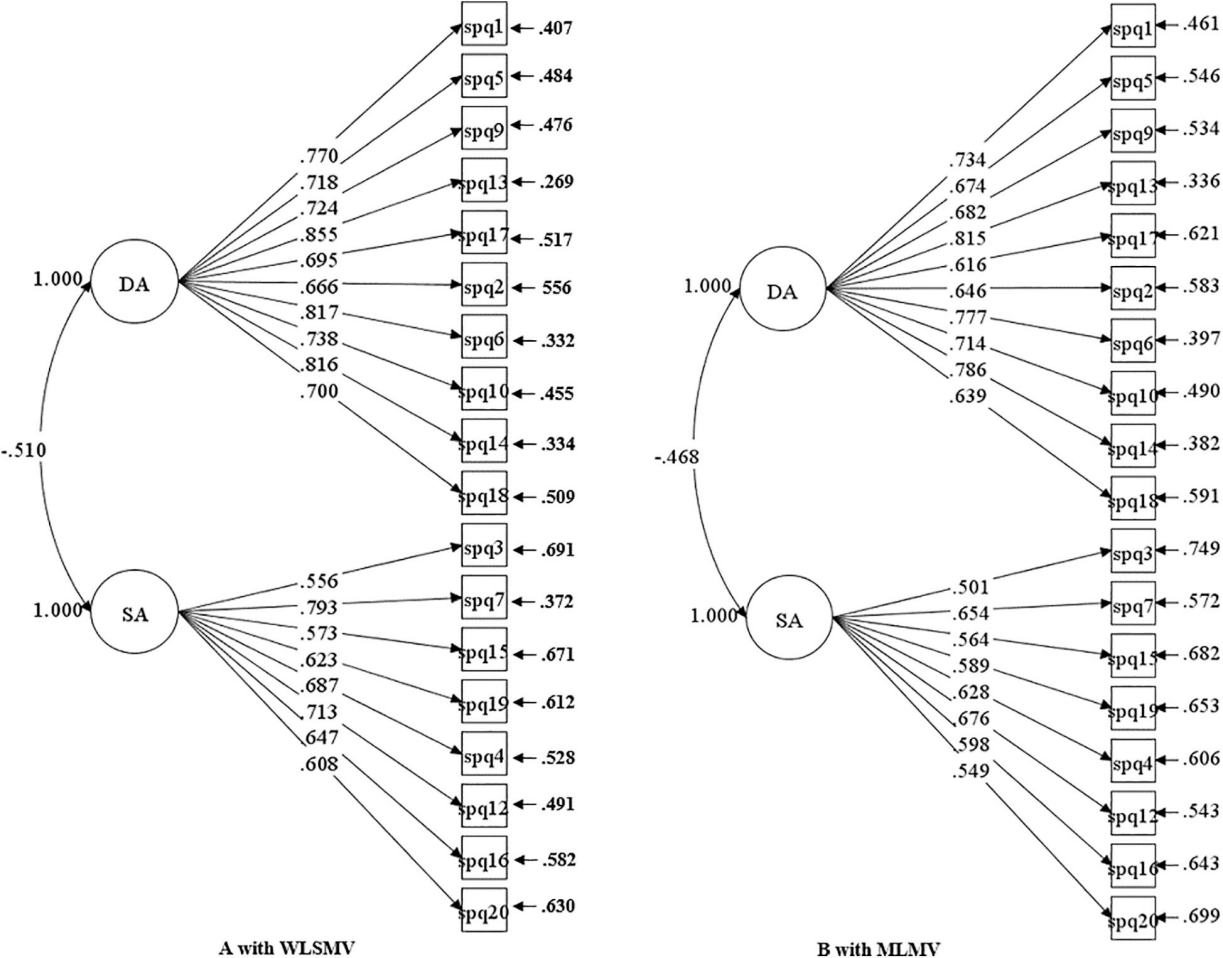
Factor structure with standardized estimated parameters of model 6.

The CFA results for Model 6 are presented in Table 5 and Figure 1.

**Table 5:** Goodness of fit indices for model 6.

Fix index	Without item 11/20 and item 8	
	WLSMV	MLMV
Chi-Square	551.229	312.925
Degrees of Freedom	134	134
Number of Free Parameters	91	55
*P*-Value	0.0000	0.0000
CFI	0.949	0.923
TLI	0.941	0.912
RMSEA	0.080	0.052
SRMR	0.048	0.049

It was evident that the removal of item 8 improved model-data fit of the two-factor model. The CFI, TLI, and SRMR values demonstrated satisfactory model adequacy, verifying the validity of Model 6. Meanwhile, the application of WLSMV estimation to Model 6 resulted in significant reductions in RMSEA and the chi-square value (*χ*²), meeting acceptable thresholds. Collectively, these indicators confirmed that the modified 18-item two-factor model possessed sound structural validity for measuring PE learning approaches among non-PE and sport undergraduates in China.

For evaluating the model's internal consistency, this study adopted the MacDonald *ω* coefficient rather than the less commonly used Cronbach *α*. Research has shown that when sample size exceeds 300, item count surpasses 5, and the factor model demonstrates good fit, MacDonald's *ω* estimates true reliability more accurately than Cronbach α (63, 64). Thus, MacDonald's ω was more suitable for this study. Furthermore, the ω coefficient accounts for each indicator's loadings and measurement error variances, enabling more accurate estimation of overall reliability. This makes it a more rigorous internal reliability measure. The calculation formula is as follows:

$$\omega =\frac{{\left(\sum \lambda i\right)}^{2}}{{\left(\sum \lambda i\right)}^{2}+\sum \theta ii}$$

The results showed that the ω reliability coefficients for the overall structure, DA subscale, and SA subscale of Model 6 were 0.947, 0.928, and 0.855, respectively. These values indicated that Model 6 had internal consistency at high (>0.8) to extremely high (>0.9) levels (56), confirming good structural reliability.

## Discussion

In the study of Biggs et al. (33), the four-factor model of the R-SPQ-2F was valid at both item and subscale levels. At the item level, all 20 items served as robust indicators for the four latent constructs: DM, DS, SM, and SS, with significant inter-construct correlations (0.93 between DM and DS, 0.70 between SM and SS, and −0.18 between DM and SM). At the subscale level, a negative correlation (−0.23) was observed between the two higher-order constructs (DA and SA), with all paths from constructs (DA and SA) to subscales (DM, DS, SM, and SS) yielding statistically significant results.

Contrastingly, the present study found that none of the tested four-factor models converged or produced admissible solutions. This finding aligned with prior research (12, 32), leading to the rejection of all four-factor configurations of the R-SPQ-2F. Instead, the first-order R-SPQ-2F with two factors of DA and SA was valid and applicable in practice. The two-factor model revealed a significant negative correlation between DA and SA (WLSMV estimate: −0.510; MLMV estimate: −0.468), which was higher than that in other similar studies (12, 32, 33, 57). This suggested the instrument demonstrated strong discriminant validity in measuring distinct learning approaches within PE contexts.

In the process of confirming the final two-factor model in this study, an item elimination strategy consistent with prior studies was adopted. Notably, the identified problematic items (items 8, 11, and 20) were precisely those subject to wording adjustments during the translation and adaptation of the R-SPQ-2F. Specifically, in the Chinese version, Items 11 and 20 were revised from Bigg's et al.'s phrasing (emphasizing rote memorization without understanding) to “practicing by rote” to align with the PE contexts. This adaptation raised questions about the appropriateness of the item modification. To clarify this doubt, kurtosis and skewness of the above three items were examined (Table 6) to analyze their data distribution characteristics and measurement equivalence.

**Table 6:** Descriptive statistical results of index variables of the R-SPQ-2F.

Item No.	Mean	Skewness	Kurtosis		Mean	Skewness	Kurtosis
SPQ1	2.939	0.298	−0.964	SPQ11	2.500	0.339	−0.724
SPQ2	2.398	0.545	−0.623	SPQ12	2.081	0.761	−0.372
SPQ3	2.823	0.122	−0.949	SPQ13	2.717	0.284	−0.787
SPQ4	2.199	0.579	−0.491	SPQ14	2.573	0.478	−0.548
SPQ5	2.900	0.171	0.412	SPQ15	2.081	0.834	0.293
SPQ6	2.632	0.412	−0.494	SPQ16	2.014	0.894	−0.034
SPQ7	1.957	1.127	0.797	SPQ17	2.258	0.750	−0.019
SPQ8	1.809	1.002	−0.012	SPQ18	2.303	0.637	−0.209
SPQ9	2.734	0.222	−0.946	SPQ19	1.783	1.195	0.973
SPQ10	2.496	0.512	−0.516	SPQ20	2.500	0.339	−0.724

The results indicated that item 8 was predominantly characterized by extremely low scores, with 51.22% of participants choosing 1: *This item is never or only rarely true of me.*), which induced extreme right-skewness in the distribution. This distribution pattern suggested poor data quality for item 8. Given that item 8 has frequently been removed in prior structural exploration of the R-SPQ-2F (12, 32, 34), this study held that the “abnormal” data did not originate from adjustments to the item description. The fundamental cause was more likely attributed to the negative connotations embedded in the original description (*I learn some things by rote, going over and over them until I know them by heart even if I do not understand them*). Such explicit negative phrasing may have consciously or unconsciously prompted participants to exhibit response avoidance.

In contrast to item 8, item 11 and 20 exhibited moderate skewness and kurtosis, with slightly right-skewed and asymmetric data distributions. Such “mild non-normality” in Likert-scale responses has been well-documented in psychological measurement literature (58–60), falling within acceptable measurement error ranges. This finding supported the methodological validity of revising the “memorization”-related phrasing in the original version to “non-comprehension-based practice” in the present study.

Regarding the redundancy of items 11 and 20, which remained unreported in prior research, this study argued that such redundancy may be attributed to the disciplinary uniqueness of PE. In most academic disciplines, the boundary between memorization-based and understanding-based learning is distinct, with students' learning approaches effectively distinguished by score differences between memory-based and application-based test items. Whereas, this boundary remains ambiguous in the discipline of PE.

Specifically, PE learning for non-PE and sports students in China centers on the “body movement”, which not only serves as the premise and foundation of learning but, more distinctively from other disciplines, assumes a dual role as both content and means. On the one hand, learners acquire knowledge about physical activities through body movement; on the other hand, their mastery of knowledge is mainly assessed through body movement. Within this dual framework of “learning about” and “learning through” body movement, “practice” emerges as the central mechanism for knowledge acquisition and assessment.

Notably, existing assessment methods for PE learning among Chinese undergraduates struggle to precisely determine whether such practice constitutes “deliberate” behavior driven by understanding (deep learning) or “mechanical” imitation and reproduction rooted in memory (surface learning). *post-hoc* random interviews on these two items revealed that participants generally focused on the “practice” behavior itself rather than its deep/surface learning attributes. Some even reported never paying attention to the way they practice in daily life, or being unclear about what needed comprehension during the learning process. This cognitive characteristic led participants to focus solely on the practice behavior when responding, overlooking other elements of the items and causing measurement redundancy in these two items.

## Limitations and prospects

First, although the samples of the present study included students from multiple majors, they were exclusively recruited from a single application-oriented university with a focus on science and engineering disciplines. To establish the general applicability of the revised R-SPQ-2F in evaluating the PE learning approaches among Chinese undergraduates, additional research is recommended across diverse institutional types.

Second, this study was a preliminary exploration of the revised R-SPQ-2F's applicability to the PE discipline, marking an initial step toward examining China's PE reform through the lens of students' learning approaches. To build on this foundation, future research could undertake two key initiatives: (1) Systematically optimizing the content and structure of the R-SPQ-2F through iterative item refinement or factor structure validation to align with the disciplinary characteristics of PE and China's educational context. (2) Adopting mixed methods (combining quantitative surveys with qualitative interviews/observations) to address questionnaire limitations, enhancing the instrument's utility and deepening insights into PE learning approaches. These efforts would strengthen the disciplinary relevance of the R-SPQ-2F and expand its capacity to inform evidence-based PE pedagogy reforms.

Third, PE as a compulsory general education course in universities is not a very common phenomenon, which limits the universality of this study to some extent. However, it is important to recognize that learning is not confined within the temporal and spatial boundaries of schools. While formal education provides a structured knowledge system, human learning constitutes a lifelong and all-around process. Informal learning contexts can also exert profound influences on knowledge acquisition and behavior shaping. Therefore, evaluating the learning quality and approaches of people's physical activity beyond the school curriculum holds significant value for global advocacy of lifelong sport to counter the modern sedentary lifestyles. Future research can adapt the R-SPQ-2F according to the goals, contents and organizational forms of different off-campus sports activities or projects, and explore the application of the instruments in a broader range of sports learning scenarios.

## Conclusion

This study ultimately validated a two-factor model for the R-SPQ-2F, encompassing 10 deep approach items and 8 surface approach items. This finding answers the core research question: within the context of Chinese education, the adapted R-SPQ-2F functions as a valid and reliable instrument for assessing the PE learning approaches of non-PE and sports undergraduates.

The findings of this study suggest that teachers and educators may utilize the R-SPQ-2F to identify the challenges students face in their approaches to PE learning. Subsequently, they can purposefully optimize teaching strategies and assessment designs, thereby improving the quality of students' PE learning by facilitating the transformation of their learning approaches. For instance, the measurement results of this study indicated that participants generally exhibited a “weak and scattered” pattern in the deep motivation (DM) and deep strategy (DS) dimensions of PE learning. The analysis showed that according to the calculation methods suggested by Biggs et al. (33), the mean scores of the DM items ranged from 2.258–2.939, with SPQ17 registering a mean of only 2.258 and displaying a positively skewed, dispersed distribution, reflecting a fragmented perception of the deep value of PE. Correspondingly, the mean scores of DS items were consistently below 2.6, and items like SPQ2 and SPQ18 presented positively skewed, platykurtic distributions, confirming that students lack actionable, systematic pathways for implementing deep strategies. In contrast, the low scores in the surface motivation (SM) and surface strategy (SS) dimensions were not due to active rejection but rather passive avoidance. This is evidenced by SM items SPQ7 and SPQ19 having means below 2.0 and displaying significantly positively skewed, leptokurtic distributions, which largely stem from social desirability bias. Furthermore, the low means for SS items SPQ8 and SPQ16 (below 2.4) highlight a critical “strategy deficit”, where mechanical practice is avoided without an effective deep strategy being utilized in its place.

Based on this precise diagnosis, the present study subsequently conducted further teaching reform experiments at the investigated universities. The new pedagogical model developed by the researchers systemically embedded interventions for motivation activation (e.g., affirming students' deep learning consciousness) and strategy implementation (e.g., providing students with specific improvement solutions to replace the mere negation of surface strategies) across the entire chain of teaching content (what to teach), teaching methodology (how to teach), and teaching assessment (how to evaluate). The experimental outcomes were favourable, demonstrating the significant effectiveness of this systemic reform rooted in the diagnosis of students' learning approaches. This practical application reinforces the value of the R-SPQ-2F as an effective instrument for guiding pedagogical design and promoting educational innovation. The detailed research procedures, explicit model construction, and quantitative analysis of the effects will be elaborated in a separate article.

## Data Availability

The raw data supporting the conclusions of this article will be made available by the authors, without undue reservation.
